# Mutability Dynamics of an Emergent Single Stranded DNA Virus in a Naïve Host

**DOI:** 10.1371/journal.pone.0085370

**Published:** 2014-01-08

**Authors:** Subir Sarker, Edward I. Patterson, Andrew Peters, G. Barry Baker, Jade K. Forwood, Seyed A. Ghorashi, Mark Holdsworth, Rupert Baker, Neil Murray, Shane R. Raidal

**Affiliations:** 1 School of Animal and Veterinary Sciences, Charles Sturt University, Wagga Wagga, New South Wales, Australia; 2 School of Biomedical Sciences, Charles Sturt University, Wagga Wagga, New South Wales, Australia; 3 Graham Centre for Agricultural Innovation (NSW Department of Primary Industries and Charles Sturt University), Wagga Wagga, New South Wales, Australia; 4 Institute of Marine and Antarctic Studies, University of Tasmania, Hobart, Tasmania, Australia; 5 Biodiversity Conservation Branch, Department of Primary Industries, Parks, Water and Environment, Hobart, Tasmania, Australia; 6 Healesville Sanctuary, Zoos Victoria, Healesville, Victoria, Australia; 7 Department of Genetics, La Trobe University, Bundoora, Victoria, Australia; George Washington University, United States of America

## Abstract

Quasispecies variants and recombination were studied longitudinally in an emergent outbreak of *beak and feather disease virus* (BFDV) infection in the orange-bellied parrot (*Neophema chrysogaster*). Detailed health monitoring and the small population size (<300 individuals) of this critically endangered bird provided an opportunity to longitudinally track viral replication and mutation events occurring in a circular, single-stranded DNA virus over a period of four years within a novel bottleneck population. Optimized PCR was used with different combinations of primers, primer walking, direct amplicon sequencing and sequencing of cloned amplicons to analyze BFDV genome variants. Analysis of complete viral genomes (n = 16) and *Rep* gene sequences (n = 35) revealed that the outbreak was associated with mutations in functionally important regions of the normally conserved *Rep* gene and immunogenic capsid (*Cap*) gene with a high evolutionary rate (3.41×10^−3^ subs/site/year) approaching that for RNA viruses; simultaneously we observed significant evidence of recombination hotspots between two distinct progenitor genotypes within orange-bellied parrots indicating early cross-transmission of BFDV in the population. Multiple quasispecies variants were also demonstrated with at least 13 genotypic variants identified in four different individual birds, with one containing up to seven genetic variants. Preferential PCR amplification of variants was also detected. Our findings suggest that the high degree of genetic variation within the BFDV species as a whole is reflected in evolutionary dynamics within individually infected birds as quasispecies variation, particularly when BFDV jumps from one host species to another.

## Introduction

Psittacine beak and feather disease (PBFD) is recognized as a key threatening process for endangered Australian psittacine birds and is a well characterized threat to a wide variety of psittacine bird species globally [1]. In affected birds PBFD typically causes immunosuppression and chronic symmetrical feather loss, as well as beak and claw deformities [2]–[6]. The disease can be expressed peracutely, ranging from sudden death, particularly in neonates [7] or as an acute form in nestling and fledglings, characterized by feather dystrophy, diarrhoea, weakness and depression ultimately leading to death within 1–2 weeks [7] or with a chronic prolonged course of feather dystrophy eventually leading to mortality [8].

The aetiological agent of the disease, *beak and feather disease virus* (BFDV) is a member of the Circoviridae family and has a relatively simple but compact circular, ambisense single-stranded DNA (ssDNA) genome of approximately 2000 nucleotides encoding a replicase (*Rep*) and a single capsid protein (*Cap*) which facilitates whole genome viral epidemiological analysis [4], [9]–[12]. Compared with other non-enveloped DNA viruses, of which the 5 kb circular genome of avian polyomavirus is probably the best benchmark, BFDV is highly genetically diverse and prone to genetic mutation, yet antigenically conserved [13]–[15]. Within Psittaciformes as a whole BFDV exhibits quasispecies characteristics with emerging geographic or host-specificity demonstrable within various clades while the observed occurrence of closely related clades in highly divergent parrot species is evidence of either host-switching or host-generalism in several BFDV lineages [16].

The last remaining wild population of the critically endangered orange-bellied parrot (*Neophema chrysogaster*) is thought to number less than 50 birds [17]–[19]. The species has been the subject of conservation efforts over the past 3 decades including the management of a captive insurance population of about 250 birds held in 3 geographically separate locations in Tasmania, Victoria and South Australia, which are used to release captive-bred birds to bolster the wild population. PBFD was recognized as a disease of concern in the first National Recovery Plan for the orange-bellied parrot [20] because the establishment of the captive-breeding program in 1985 was set back by an outbreak of the disease. However, the use of improved facilities and routine testing for infection by PCR, hemagglutination and hemagglutination inhibition (HI) assays has been used successfully between 1994 and 2006 to manage and prevent the transmission of infection in the captive flock.

Extensive PCR and serological testing in 2006 revealed no evidence of BFDV infection in the Tasmanian captive flock of orange-bellied parrots [21]. However, in the summer breeding season of 2007–08 several juvenile birds in the Tasmanian captive flock developed clinical signs of PBFD which was confirmed by laboratory testing. A decision was made to test all birds in the captive breeding programme and to sample the wild population in order to determine the source and extent of the infection. At this time, of 132 Tasmanian birds tested 35 were PCR positive with blood samples and a further 3 birds were positive with feather samples (28.8% PCR positive). HI antibody titres ranging from 1∶20 to 1∶2,560 were detected in 47 of 132 (35.6%) samples tested. In total 71/132 (53.8%) birds in the Tasmanian flock had laboratory evidence of current (PCR positive) or recent (HI positive) BFDV infection. Three birds that were PCR positive also had clinical signs of PBFD and high feather HA results (titres ranging from 1∶80 to 1∶10,240), indicating viral shedding. Similarly, of 71 birds tested in the Victorian flock 6 were PCR positive and a further 3 birds were HI positive and most of these had a history that included translocation from Tasmania and/or South Australia. Of the 20 birds in the South Australian flock 2 were PCR positive. Efforts to control the disease included euthanasia of clinically diseased birds and segregation of clinically normal but PCR positive or HI positive birds and serial retesting. The recent detection of two distinct lineages of BFDV in the remnant wild population of orange-bellied parrots, consisting of fewer than 50 birds, suggests a role for other parrot species as a reservoir for infection by spillover into this critically endangered species [22]. Given the overall small population size of the captive flock and lack of any immediately previous evidence of endemicity this almost certain cross-species infection provided a unique scenario to track longitudinally the evolution of BFDV replication in a well characterized novel host with well documented health status.

## Materials and Methods

### Sampling and Extraction of Genomic DNA

Samples used in the analyses described below were obtained during the course of normal health monitoring and veterinary checks of birds held in captive breeding flocks controlled by the Tasmanian Government Biodiversity Conservation Branch, and the Victorian Government controlled Healesville Sanctuary, Zoos Victoria Australia. Animal sampling was obtained using guidelines set by the Australian Code of Practice for the Care and Use of Animals for Scientific Purposes (1997) and authorized by the Charles Sturt University Animal Research Authority (permit 09/046). No additional suffering or discomfort was endured by any animals referred to in this manuscript. BFDV sequences were amplified from dried blood samples collected directly onto filter paper from 35 orange-bellied parrots that were previously tested positive for BFDV infection by the Veterinary Diagnostic Laboratory (VDL), Charles Sturt University, using established methods [23], [24]. Archived samples included samples from the captive orange-bellied parrot flocks in Victoria and Tasmania. For extraction of total genomic DNA, three spots of blood each approximately 5 mm in diameter were cut out using scalpels according to the methods described by Bonne et al. [25] and collected in a microcentrifuge tube (Eppendorf), and DNA was extracted with the Qiagen blood mini kit (Qiagen, Germany), using a modified dried blood spot protocol.

### PCR Amplification, Cloning and Sequencing of BFDV Genome

Published BFDV genome sequences were aligned with Geneious Pro 6.1.6 (Biomatters, New Zealand) in order to identify conserved regions and design primers. Initially, PCR was conducted targeting 717-bp section of the *Rep* gene identical to published methods [24] and all of the positive samples were sequenced by the Australian Genome Research Facility Ltd (AGRF Ltd., Brisbane), using a Sanger-based AB 3730×l unit (Applied Biosystems) with the same primers from the PCR. A number of other primer sets (Table 1) were used to obtain full genome amplification and sequencing of BFDV from 16 positive orange-bellied parrots. Reactions for different primer combinations were optimized, and the optimized reaction mixture contained 3 µl extracted genomic DNA, 2.5 µl of 10× High Fidelity PCR Buffer (Invitrogen), 1 µl of 25 µM of each primer, 1 µl of 50 mM MgSO_4_, 4 µl of 1.25 mM dNTP’s, 1 U platinum® *Taq* DNA Polymerase High Fidelity (Invitrogen) and DEPC water added to a final volume of 25 µl. The optimized reaction was run as follows: 95°C for 3 min, followed by 40 cycles of 95°C for 30 s, 57°C for 45 s and 68°C for 2 min, and finally 68°C for 5 min. The extension time for the second and fourth sets of primer combinations were 30 s and 90 s respectively instead of 2 min. In each set of reactions, BFDV genomic DNA and distilled H_2_O were included as positive and negative controls, respectively.

**Table 1:** Details of primer used in this study in different combinations to amplify the full genome of BFDV DNA*.

Primer	Primer sequence	bp position	Reference
1	5′-GTTATACGCCGCCGTAATC-3′	84–102	Ypelaar et al. (1999)
2	5′-AACCCTACAGACGGCGAG-3′	182–199	Ypelaar et al. (1999)
4	5′-GTCACAGTCCTCCTTGTACC-3′	879–898	Ypelaar et al. (1999)
BFDV-B-R	5′-AGCCCTCCTTGGACGGC-3′	151–167	Designed in this study
BFDV-C-R	5′-CGTCCAACGATGGCATAGT-3′	255–273	Designed in this study
BFDV-I-F	5′-GCAAACTGACGGAATTGAACATA-3′	1309–1330	Designed in this study
BFDV-J-R	5′-TTGGGTCCTCCTTGTAGTGG-3′	1422–1441	Designed in this study

Combinations attempted were 2 and BFDV-B-R; 1 and BFDV-C-R; 2 and BFDV-J-R; BFDV-I-F and BFDV-C-R.

The resulting PCR products were separated on a 0.8% agarose gel, and the appropriate bands were excised and purified using the Wizard® SV Gel and PCR Clean-Up System (Promega, USA) according to the manufacturer’s instructions. Purified amplicons were cloned using pGEM®-T Easy Vectors (Promega, USA) and recombinant plasmids were purified using a PureYield™ Plasmid Miniprep System (Promega, USA) according to the manufacturer’s instructions. Purified inserts were sequenced at least twice in each direction with M13 forward and reverse primers as well as some suitable internal primers by AGRF Ltd. as described above. The sequences were trimmed for vector, aligned to construct contigs using a minimum overlap of 35 bp and a minimum match percentage of 95%, and constructions of full genome sequence were carried out in Geneious Pro 6.1.6 (Biomatters, New Zealand) and BioEdit Sequence Alignment Editor (version 7.1.6.0).

### Sequence Analysis

The sequences were aligned in Geneious (version 6.1.6, Biomatters, New Zealand) using the ClustalW (gap open cost = 10; gap extension cost = 5) [26], but no insertion or deletion were inferred from the alignments. Bayesian phylogenetic trees and the evolutionary rate were inferred using the program Beast v1.7.5 [27]. Two independent Monte Carlo-Markov chains (MCMC) were implemented for the full genome and partial *Rep* gene data sets separately, with 200,000,000 iterations under a range of different nucleotide substitution models and tree priors with a thinning of 20,000. The Bayesian skyline coalescent demographic prior was chosen because it allows temporal changes in population size [28]. Each analysis was checked to ensure that a reasonable effective sample size (ESS>200) had been reached for all parameters. For the full genome and partial *Rep* gene dataset a general-time-reversible model with gamma distribution rate variation and a proportion of invariable sites (GTR+I+G4) was identified using program jModelTest 2.1.3 [29]. Tracer version v1.5 was used to derive parameters and TreeAnnotator v1.7.5 was used to obtain the tree with the highest clade credibility and posterior probabilities for each node [27], as well as FigTree v1.4 was used to generate the consensus tree [30]. The evolutionary rate was inferred under both relaxed (uncorrelated exponential and uncorrelated lognormal) and strict molecular clock.

We screened for evidence of recombination amongst BFDV genomes using the program SBP and GARD [31] under a range of nucleotide substitution models and site-to-site rate variation on the Datamonkey webserver [32], and DualBrothers in Geneious 6.1.6 [33], [34]. We also used the GENECONV [35], Bootscan [36], Chimaera [37], Siscan [38] and RDP [39] methods contained in the RDP4 program [40]. Events that were detected by at least three of the aforesaid methods with significant *p*-values were considered plausible recombinant events.

To discover evidence of positive selection sites in the protein coding genes of BFDV from orange-bellied parrot (where *ω*, the selection parameter which corresponds to the ratio of the nonsynonymous and synonymous substitution rates, is greater than 1), a number of methods were used. A Fast Unconstrained Bayesian AppRoximation (FUBAR) was used, which allows sites experiencing positive and purifying selection [41], where the number of grid points was 400. This compared the number of nonsynonymous and synonymous substitution in typical random effects approaches. The data were further analyzed in the programs FEL and SLAC [42] on the Datamonkey webserver to detect positive selection (initially data were screened for recombination). Finally, mixed effects model of evolution (MEME) was used to screen for episodic positive selection [43], which is capable of identifying sites where a proportion of branches have evolved under positive selection.

To investigate epistasis on the joint distribution of the substitution events among positions in the protein coding sequence, Bayesian Graphical Model (BGM) [44] was used on the Datamonkey webserver, where each branch in the phylogeny is a unit of observation.

## Results

### Analysis of BFDV DNA Sequences from Orange-bellied Parrots

**Figure 1:**
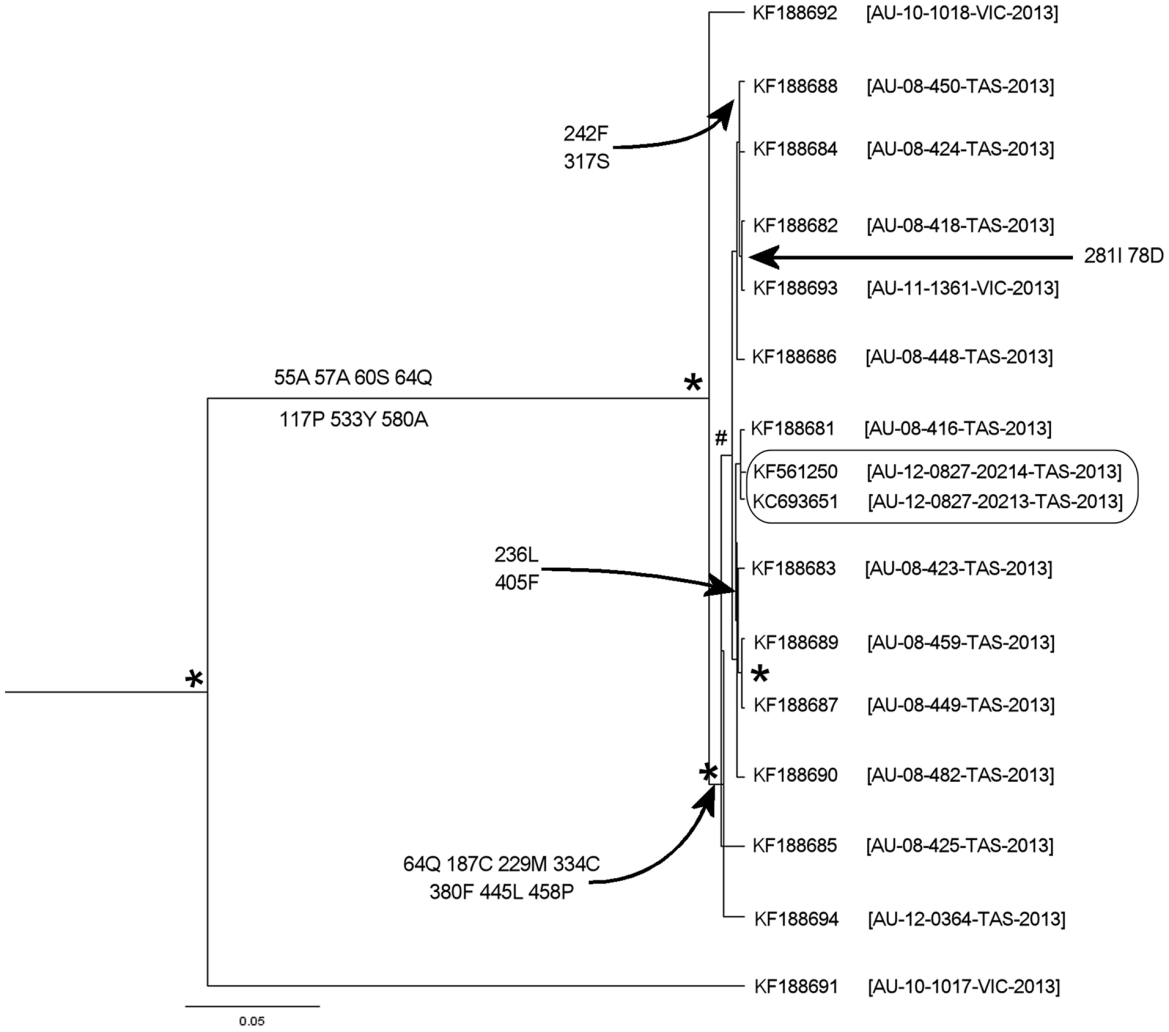
Bayesian phylogenetic tree inferred evolutionary relationships among BFDV full genome sequences from orange-bellied parrots. Maximum clade credibility tree automatically rooted by using relaxed molecular clock model in Beast v1.7.5. Labels at branch tips refer to GenBank accession number, and with country code, original sample ID, collection site and year of isolation in parentheses. Nodes with posterior probability of ≥0.95 are indicated with asterisks and with a hash for *P*≥0.7. Inferred nonsynonymous substitutions at codons are indicated at the appropriate lineages where the majority of genomes in a clade possess a particular substitution then the ancestral node has been labeled.

Phylogenetic analysis of entire BFDV genome sequences revealed two distinct and unique BFDV genotypes in the orange-bellied parrot flock. The first genotype (OBP-I, GenBank accession numbers: KC693651, KF561250, KF188681–KF188690 and KF188692–KF188694) shared >99% pairwise both for nucleotide and amino acid identity with each other, whereas the second genotype (OBP-II, GenBank accession number: KF188691) shared >90% and >82% pairwise nucleotide and amino acid identity respectively with all other orange-bellied parrot BFDV sequences. These two genotypes had no significant relationship with other BFDV sequences available from GenBank (Figure S1 and Table S1). To examine the mutations that may have been responsible for the outbreak, we constructed phylogenetic trees using the entire genome and partial *Rep* gene sequences. Full genome analysis are shown in Figure 1 and depict 42 mutations, 19 of which are nonsynonymous substitutions while the remaining 23 are synonymous substitutions (the frequency histogram of the mean coefficient variation; mean CoV = 1.24). Two different haplotypes ([55A,57A,60S,64Q,117P,533Y,580A] and [64Q,187C,229M,334C,380F,445L,458P]) were detected from 19 nonsynonymous mutations in the 15 orange-bellied parrots BFDV genomes (Figure 1).

**Figure 2:**
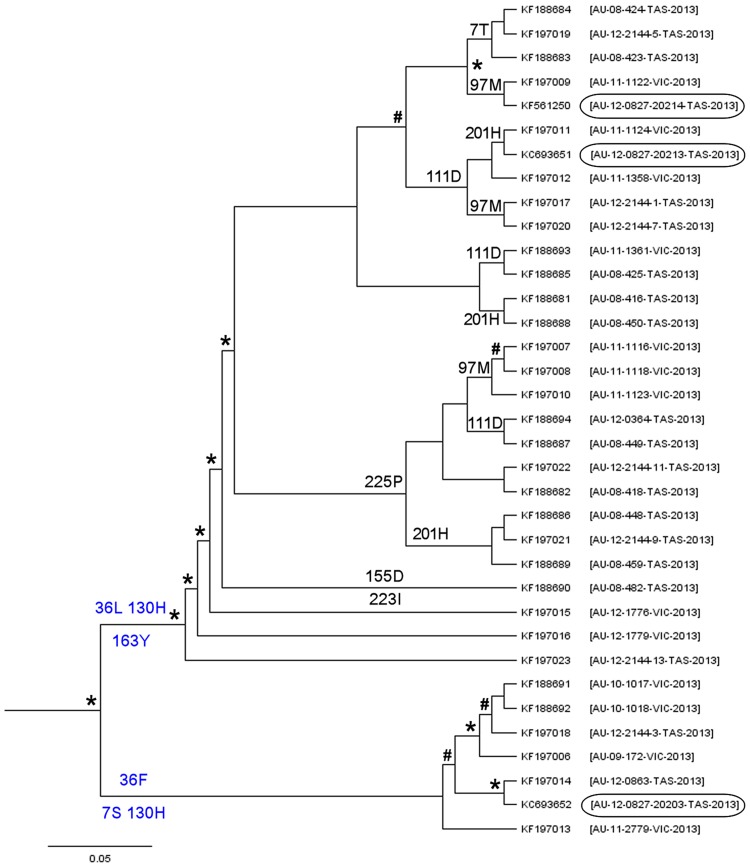
Bayesian phylogenetic inference of evolutionary relationship among *Rep* gene sequences from orange-bellied parrots. Maximum clade credibility tree automatically rooted by using relaxed molecular clock model in Beast v1.7.5. Labels at branch tips refer to GenBank accession number, and with country code, original sample ID, collection site and year of isolation in parentheses. Nodes with posterior probability of ≥0.9 are indicated with asterisks and with a hash for *P*≥0.6. Inferred nonsynonymous substitutions (blue colour) at codons are indicated at the appropriate lineages where the majority of genomes in a clade possess a particular substitution then the ancestral node has been labeled.

Independent analysis of 35 *Rep* gene sequences revealed 18 mutations, 5 of which were nonsynonymous substitutions (Figure 2), with one at codon 36 in a rolling-cycle replication motif [45] a substitution from phenylalanine to leucine. The pattern of changes are explained by 2 different haplotypes at [36L,130H,163Y] and [7S,36F,130H] representing the majority of the outbreak.

### Positive Selection in the BFDV Genome

Positively selected sites inferred by FUBAR, FEL, SLAC and MEME are shown in Table 2. These analyses indicated 19 sites under positive selection within the entire genome of orange-bellied parrot BFDV genomes, although SLAC detected only 5 codon sites. Independent analysis of the *Rep* gene sequences failed to consistently demonstrate sites under positive selection by all four methods. For both the entire orange-bellied parrot BFDV genome data and the *Rep* gene data we did not detect any significant co-evolving site by BGM analysis.

**Table 2:** Positively selected sites in BFDV from orange-bellied parrots inferred by different methods.

Gene	No. ofsequences	Codon (s)			
		FUBAR (*ω*; *P^a^*)	FEL (*ω*; *P^b^*)	SLAC (*ω*; *P^c^*)	MEME (*P^d^*)
Fullgenome	16	55 (4.10; 0.76), 57 (4.10; 0.76),60 (4.53; 0.76), 117 (10.52; 0.93),187 (3.92; 0.75), 218 (2.57; 0.73),229 (2.73; 0.73), 445 (6.78; 0.90),242 (3.59; 0.73), 334 (4.27; 0.77),380 (3.97; 0.75), 445 (6.76; 0.90),458 (4.42; 0.77), 533 (4.07; 0.78),580 (8.96; 0.92)	55 (∞; 0.33), 57 (∞; 0.33), 60 (∞; 0.23),117 (∞; 0.14), 187 (∞; 0.31), 218 (∞; 0.24),229 (∞; 0.39), 242 (∞; 0.36), 317 (∞; 0.50),334 (∞; 0.33), 380 (∞; 0.32), 445 (∞; 0.21),533 (ω; 0.35), 458 (∞; 0.23), 580 (∞; 0.16)	117 (11.10; 0.44), 445 (11.03; 0.45), 458 (10.54; 0.46), 533 (10.57; 0.10),580 (10.27; 0.23)	55 (0.08), 57 (0.08), 60 (0.06), 64 (0.03), 78 (0.05), 117 (0.16), 187 (0.11), 218 (0.04), 229 (0.13), 236 (0.08), 242 (0.06), 317 (0.06), 334 (0.09), 380 (0.09), 405 (0.07), 445 (0.23), 458 (0.09), 533 (0.34), 580 (0.19)
*Rep*	35	7 (2.32; 0.76), 130 (2.26; 0.75),163 (4.57; 0.87)	7 (∞; 0.19), 163 (∞; 0.02)	No sites	7 (0.21), 155 (0.09), 130 (0.02), 163 (0.001)

Estimates of ω represents the selection parameter and *P^a^* the posterior probability, and *P* ^b,c,d^ the level of significance from the posterior probability of ω>1 at a site derived from the approximate codon model.

### Recombination Analysis

**Figure 3:**
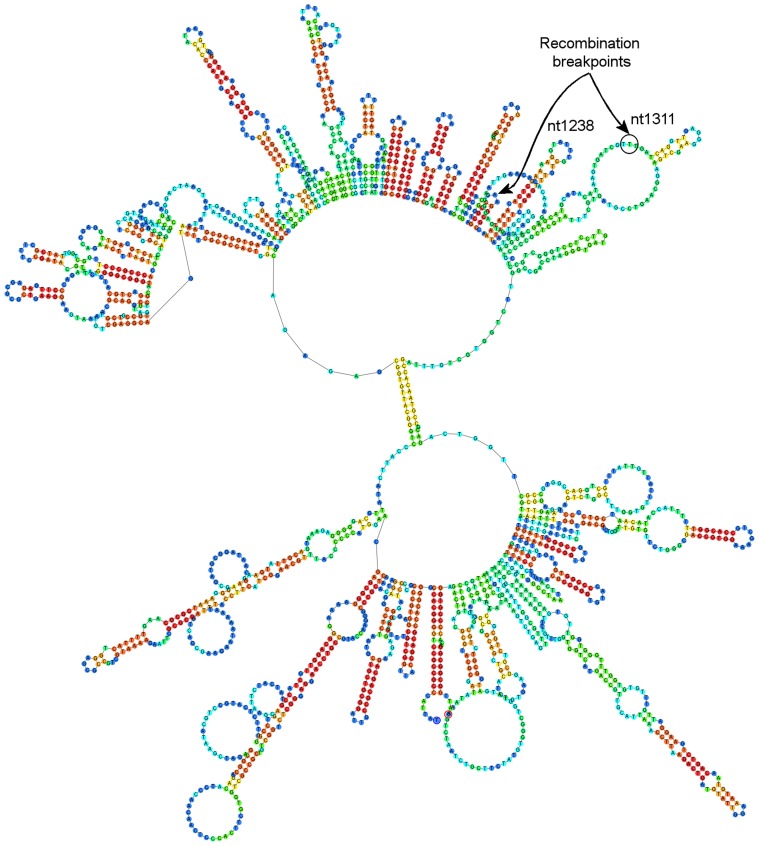
DNA folds analysis for demonstrating loop-like DNA structure within recombination breakpoint locations. Predicted DNA fold analysis showing recombination breakpoint locations within loop structures in the BFDV genome (12-0827-201213, GenBank accession: KC693651) [22] using tools available in Geneious 6.1.6. The first recombination breakpoint location at thymidine nt location 1311 (*P*≤0.001) as circled was a consistently predicted loop structure in all recombinant genomes (n = 15), a second recombination breakpoint location at 1238 (*P*≤0.05) is also shown (arrow) in a smaller loop structure. Colors of nucleotides represent base-pair probabilities (red = high, green = mid, blue = low).

Using SBP, GARD, DualBrothers and RDP4 recombination was detected in the BFDV genomes (n = 16). The strongest support for recombination was detected in the c-terminal portion of the capsid gene between two BFDV genomes that were from geographically distant birds. One (11–1361) was a captive bird from Victoria and the second (12-0827-20213) was a Tasmanian bird originally sourced from the wild. A second recombination in the intergenic region of the genome was also strongly supported between another wild-caught BFDV (12-0827-20214) and a captive-bred (08-423) BFDV found in the Tasmanian flock. DNA folding analysis revealed that these recombination breakpoints were consistently predicted to be physically located within loop structures (Figure 3) of the encapsidated genome. Independent analysis of sequenced *Rep* gene sections from 35 orange-bellied parrot BFDV genomes revealed one significant recombination event between wild (12-0827-20203) and captive (12-1776) orange-bellied parrot BFDV from Tasmania and Victoria respectively [breakpoint location: 618; *P≤*0.001].

### Estimation of Evolutionary Rates of BFDV

In Beast a variety of different demographic clock models were supported but the mean evolutionary rate was better fitted by a relaxed molecular clock than a strict molecular clock. With a relaxed molecular clock the mean evolutionary rate estimated for the full genome was 3.41×10^−3^ subs/site/year (95% HPD: 3.43×10^−4^ to 8.08×10^−3^) and the mean evolutionary rate in the *Rep* gene data set was much lower than that for the entire genome, 3.22×10^−6^ subs/site/year (95% HPD: 2.38×10^−7^ to 1.55×10^−5^).

### Sequences Analysis of BFDV Genome and its Quasispecies Variants

**Figure 4:**
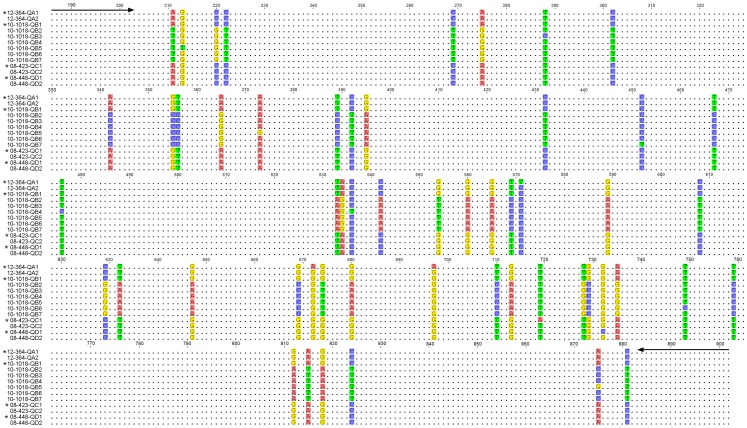
Alignment of 13 *Rep* sequences showing BFDV quasispecies variants in orange-bellied parrots. The variants 12-364QA1 and 12-364QA2 (GenBank accession numbers: KF188694 and KF188695 respectively) originated from a single bird, 10-1018-QB1 to 10-1018-QB7 (GenBank accession numbers: KF188692, KF188696-KF188701 respectively) originated from another orange-bellied parrot (10-1018), 08-423CQ1 and 08-423CQ2 (GenBank accession numbers: KF188683 and KF188702 respectively) represented orange-bellied parrot 08-423, while 08-448-QD1 and 08-448-QD2 (GenBank accession numbers: KF188686 and KF188703 respectively) originated from another orange-bellied parrot. For each individual BFDV sequence, asterisks indicate those where full genome (1993 bp) sequences were performed as well and supported by at least 8 clones. Variants from these (suffixes 2–7) came from multiple sequencing of PCR amplicons directly as well as cloned products. From Table 1 the primer 2 forward (5′-AACCCTACAGACGGCGAG-3′) and 4 reverse (5′-GTCACAGTCCTCCTTGTACC-3′) are indicated by arrows.

In the sample from one orange-bellied parrot (10–1018) preferential amplification of one genotype (10-1018-QB1 in Figure 4 and GenBank accession number: KF188692) was detected using primer set 2 and BFDV-B-R (Table 1). This primer set produced an amplicon of 1962 nucleotides which was cloned and sequenced 8 times separately, producing the same sequence data (KF188692). On the other hand, by analyzing 16 separate clones, multiple variants (n = 6) were detected in the same bird when using primer set 2 and 4, which produces a *Rep* gene amplicon of 717 nucleotides (Figure 4). In this bird the second primer set failed to amplify the first genotype even though the target sequences were identical, however there was a T-A substitution at site 211 which is close (7 nucleotides) to the binding site for primer 2 and also a C-T substitution at site 881 which is only one nucleotide from the binding site of the reverse primer 4 (Figure 4).

Further cloning and sequencing of samples from three orange-bellied parrots (12-364, 08-423 and 08-448) demonstrated two important quasispecies variants in each bird (Figure 4 and Table 3). Within one of these birds (10-1018), and out of a total of seven variants, 10-1018-QB1 differed significantly (>93% pairwise nucleotide and amino acid identity) with all other neighbor variants. Other variants within the same bird had >99% pairwise nucleotide similarity. Variant 10-1018-QB5 had three mutations, two of which were nonsynonymous substitutions. The first mutation gave rise to a phenylalanine residue from valine at codon 10, and the second substitution was from lysine to arginine at codon 63.

**Table 3:** Percentage identity of quasispecies variants of orange-bellied parrot isolates.

	Nucleotide identity (%)												
variantsQuasispecies	12-364-QA1	12-364-QA2	10-1018-QB1	10-1018-QB2	10-1018-QB3	10-1018-QB4	10-1018-QB5	10-1018-QB6	10-1018-QB7	08-423-QC1	08-423-QC2	08-448-QD1	08-448-QD2
**12-364-QA1**	–	98.7	98.7	93.3	93.3	93.3	92.5	93.3	93.3	98.3	98.7	98.3	98.7
**12-364-QA2**	99.6	–	100	94.6	94.6	94.6	93.7	94.6	94.6	99.6	100	99.6	100
**10-1018-QB1**	99.6	100	–	94.6	94.6	94.6	93.7	94.6	94.6	99.6	100	99.6	100
**10-1018-QB2**	93.7	94.1	94.1	–	100	100	99.2	100	100	94.1	94.6	94.1	94.6
**10-1018-QB3**	93.6	94.0	94.0	99.9	–	100	99.2	100	100	94.1	94.6	94.1	94.6
**10-1018-QB4**	93.4	93.9	93.9	99.7	99.6	–	99.2	100	100	94.1	94.6	94.1	94.6
**10-1018-QB5**	93.4	93.9	93.9	99.6	99.4	99.3	–	99.2	99.2	93.3	93.7	93.3	93.7
**10-1018-QB6**	93.6	94.0	94.0	99.9	99.7	99.6	99.4	–	100	94.1	94.6	94.1	94.6
**10-1018-QB7**	93.6	94.0	94.0	99.9	99.7	99.6	99.4	99.7	–	94.1	94.6	94.1	94.6
**08-423-QC1**	99.4	99.9	99.9	94.0	93.9	93.7	93.7	93.9	93.9	–	99.6	99.2	99.6
**08-423-QC2**	99.6	100	100	94.1	94.0	93.9	93.9	94.0	94.0	99.9	–	99.6	100
**08-448-QD1**	99.4	99.9	99.9	94.0	93.9	93.7	93.7	93.9	93.9	99.7	99.9	–	99.6
**08-448-QD2**	99.6	100	100	94.1	94.0	93.9	93.9	94.0	94.0	99.9	100	99.9	–
	**Amino acid identity (%)**												

Although the number of mutations in these three birds (12-364, 08-423 and 08-448) was not high, they were nonsynonymous substitutions which could therefore result in structurally significant alterations and functionality of *Rep*. Variants 12-364-QA1 and 12-364-QA2 showed 98.7% nucleotide identity with three nonsynonymous substitutions which are also likely to modify the *Rep* structure. The first mutation gave rise to a leucine residue from serine at codon 129, the second mutation was from glycine to arginine at codon 163, and the third mutation occurs at codon 171 and was a substitution from glycine to aspartic acid.

Another four variants from two different birds (08-423 and 08-448) experienced similar (>99%) pairwise nucleotide and amino acid identity. Variants 08-423-QC1 and 08-423-QC2 had one nonsynonymous substitution at codon 178 which was a substitution from aspartic acid to glutamic acid whereas variants 08-448-QD1 and 08-448-QD2 had a substitution giving rise to leucine residue from valine at codon 183.

## Discussion

This paper describes a longitudinal study of quasispecies variants and recombination events in an emergent outbreak of BFDV infection in a naïve population of the critically endangered orange-bellied parrot. Whilst the origins of the BFDV infections in this population of birds are unknown it is almost certain to have been another psittacine bird species, presumably from the wild in Tasmania, and not from within the orange-bellied parrot population given its small size, as well as the pre-existing health monitoring that was in place immediately before the outbreak of infection that indicated an absence of both viremic birds and circulating antibodies to BFDV. This study is the first to show the importance of whole genome versus partial gene analysis for characterizing the evolutionary pathway of a ssDNA virus recently infecting a naïve host species.

Even though it represents almost 40% of the entire viral genome, analysis of the *Rep* gene alone was not satisfactory for interrogating the evolutionary dynamics of the infection. As shown by whole genome analysis in BFDV [16] and other viruses [46], [47], our results comparing *Rep* gene (n = 35) and the whole genome (n = 16) support the latter as more robust for detecting recombination as well as phylogenetic and evolutionary studies. Phylogenetic analysis of the full BFDV genomes revealed one haplotype dominated by codons within the *Rep* gene and, in contrast to Kundu et al. [48], a second haplotype dominated by codons within the *Cap* gene (Figure 1). This indicates that change in not only functionally important *Rep* but also in immunogenic *Cap* was associated with the outbreak of BFDV infection in orange-bellied parrots. Different factors may add to the scope and rapidity of an adaptive sweep that might increase the fitness of a mutated haplotype, including the environmental stability of circoviruses [49], modes of both horizontal and vertical transmission [50]–[52] as well as host population dynamics.

Our analyses consistently demonstrated evidence of positive and episodic selection at the level of an individual site (Table 2) that were linked with outbreak haplotypes (Figure 1) in the case of full genome sequences. However, of the methods used, FUBAR, MEME and FEL were superior to SLAC. Even though many more sequences were available for analysis (n = 35) the results from studying *Rep* gene alone were inadequate for detecting positive and episodic sites suggesting that these methods may be inappropriate in shorter sequences for detecting rare adaptive sweep. This may be the reason why Kundu et al. and Heath et al. failed to demonstrate consistent evidence of positively selected sites in BFDV [9], [48]. In general, many of the site prediction methods that underpin such analyses have limitations [53] with several approaches suggesting that accuracy and power is better with longer sequences [41], [42], [54]. Some of these require considerable sequence divergence as well as a substantial number of taxa [54]. The majority of the polymorphic sites documented in Figure 1 and Figure 2 suggest that both functionally important *Rep* and immunogenic *Cap* was associated with BFDV evolution in orange-bellied parrots.

During the course of infection numerous BFDV genome variants were present in the orange-bellied parrot flock and within individual birds. To confirm quasispecies variants high fidelity *Taq* DNA polymerase was used which has an error-correcting mechanism [55]. With the exception of one highly divergent variant 10-1018-QB1 (Figure 4), which could be considered as a dual infection with a separate genotype altogether, other variants were relatively close with 98% similarity. In the only other study of naturally occurring ssDNA viral quasispecies a novel unassigned circovirus-like infection in sea turtles consistently showed up to 5 variants, with the majority being >80% similar, with occasional extremely divergent variants within individual animals [56]. Virus quasispecies events are not limited to ssDNA or ssRNA virus evolutionary dynamics. Recently, others reported that there were seven different genotypes of tick-transmitted bacterium *Borrelia afzelii* in the bank vole *Myodes glareolus* which favors greatly antigenic diversity [57]. Similarly in protists such as the agent of human malaria, *Plasmodium falciparum,* up to 5 different strains may be detected simultaneously within individuals [58] and this presumably is a key influence on the evolution of virulence [59].

Preferential PCR amplification of different BFDV genotypes was detected within an individual animal (Figure 4). Others have shown that this can result from significant GC% differences between sequences if the denaturation, salt and co-solvent conditions of the reaction favour one genotype over another or if the PCR products differ in length, especially if the larger target DNA is degraded [60]. However, these scenarios are unlikely to be the cause in our situation. Stochastic fluctuation in the number of copies of each target sequence can result in what appears to be preferential amplification when the initial number of templates is very small [60]. As shown in Figure 4 it is more likely that there was less efficient priming of DNA synthesis of one target versus another because of immediate downstream mismatches close to the primer binding site resulting in preferential amplification of the other sequence.

High mutation rates resulting in quasispecies dynamics lead to interactions on a functional level and may be the major contributing factor in the adaptability of RNA viruses to changing environments [61], [62]. This was most likely occurring in orange-bellied parrots as we detected high evolutionary rates consistent with those of other BFDV lineages [9], [48] and other small ssDNA viruses [28], [55], [63]–[67], and approaching that for RNA viruses whose polymerase lacks proofreading ability [55], [68]. Indeed, the simultaneous occurrence of recombination and relatively lower mutation rates in the *Rep* data set where recombinants were included reveals that this process has significant effects on estimation [69]. The high level of sequence diversity among the BFDV genomes support the theory that evolutionary rates in viruses are not simply a consequence of high fidelity polymerase activity but also controlled by genomic architecture and replication speed [55].

Viral recombination and point mutations are key evolutionary mechanisms driving pathogen diversity and host adaptation [70]–[73] and, in the case of influenza A virus, have been shown to occur between pathogen and host as a mechanism for acquiring virulence [74]. Sequences from ancestral ssDNA viruses have been found in vertebrate genomes suggesting that parvoviruses and circoviruses have been present for at least 40 million years [75]. Recent global analysis of BFDV as well as other ssDNA viruses [13], [16], [76], [77] predicted two significant recombination hotspots in BFDV, one in the c-terminal portion in the coat protein, and a second in the intergenic region of the genome and our results provide strong natural evidence of this. Furthermore the c-terminal recombination breakpoint was consistently (n = 15) within loop-like structures predicted by DNA fold analysis as shown in Figure 3. In site-specific genetic recombination within prokaryotic and eukaryotic genes, DNA looping is an important mechanism when synonymous sites are close to one another at the time of strand exchange [78]. The loop-like DNA structures in BFDV may represent an important recombination site for circoviruses which replicate via rolling circle replication. This process is dependent on a highly conserved stem-loop structure located immediately before the *Rep* gene which provides the replication binding site for *Rep*. Within a dual-infected cell recombination may be more permissible during viral uncoating in its ssDNA form or during interactive rolling circle replication as ssDNA molecules are produced.

## Supporting Information

Figure S1 **Outgroup-rooted Maximum-likelihood phylogenetic inference of evolutionary relationships among BFDV genome sequences.** ML tree was constructed using BFDV full genome sequences from orange-bellied parrots with publicly available full-length BFDV genomes (see Table S1 for more details) with 1000 bootstrap resamplings and a raven circovirus (GenBank accession: DQ146997) as outgroup. Blue color indicates the orange-bellied parrot genotype-I (OBP-I) and red color indicates the orange-bellied parrot genotype-II (OBP-II).
(TIF)

Table S1 **BFDV full genome sequences used for discovering evolutionary pathway in the orange-bellied parrot.**
(DOC)
