# Supplementary figures and images for: Mutability Dynamics of an Emergent Single Stranded DNA Virus in a Naïve Host
Source: PLoS One. 2014 Jan 8;9(1):e85370. doi: 10.1371/journal.pone.0085370 (PMC3885698; doi:10.1371/journal.pone.0085370)

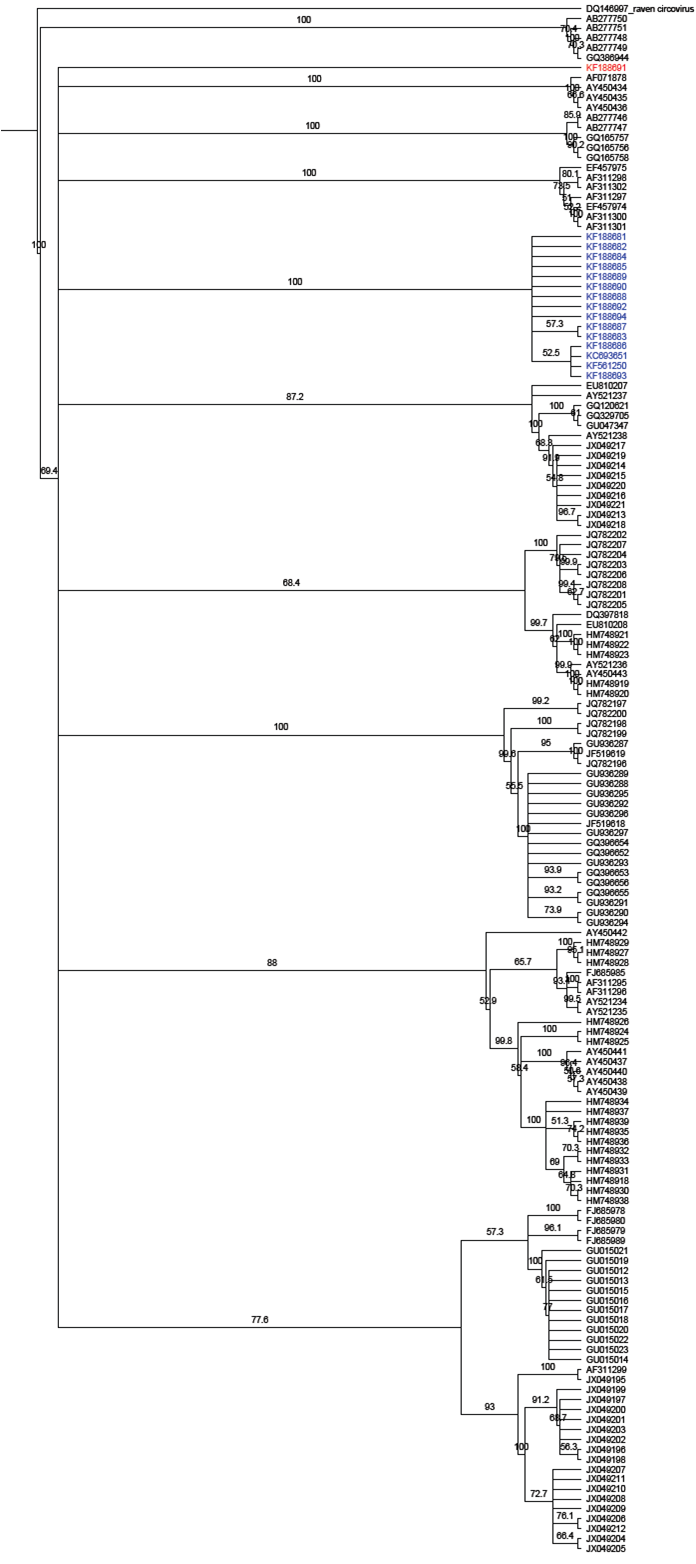

Supplement: Figure S1 — Outgroup-rooted Maximum-likelihood phylogenetic inference of evolutionary relationships among BFDV genome sequences. ML tree was constructed using BFDV full genome sequences from orange-bellied parrots with publicly available full-length BFDV genomes (see Table S1 for more details) with 1000 bootstrap resamplings and a raven circovirus (GenBank accession: DQ146997) as outgroup. Blue color indicates the orange-bellied parrot genotype-I (OBP-I) and red color indicates the orange-bellied parrot genotype-II (OBP-II). (TIF) [file pone.0085370.s001.tif]
