# Supplementary material for: Mutability Dynamics of an Emergent Single Stranded DNA Virus in a Naïve Host
Source: PLoS One. 2014 Jan 8;9(1):e85370. doi: 10.1371/journal.pone.0085370 (PMC3885698; doi:10.1371/journal.pone.0085370)
Supplement: Table S1 — BFDV full genome sequences used for discovering evolutionary pathway in the orange-bellied parrot. (DOC) [file pone.0085370.s002.doc]

**Table S1: BFDV full genome sequences used for discovering evolutionary pathway in the orange-bellied parrot**

| **Accession number** | **Country** | **Year of Isolation** | **Common name** | **Host Species** | **Wild/Captive** | **Reference** |
| --- | --- | --- | --- | --- | --- | --- |
| KC693651 | Australia | 2013 | Orange-bellied parrot | *Neophema chrysogaster* | Wild | Peters et al., 2013 |
| KF188681 | Australia | 2013 | Orange-bellied parrot | *Neophema chrysogaster* | Captive | This study |
| KF188682 | Australia | 2013 | Orange-bellied parrot | *Neophema chrysogaster* | Captive | This study |
| KF188683 | Australia | 2013 | Orange-bellied parrot | *Neophema chrysogaster* | Captive | This study |
| KF188684 | Australia | 2013 | Orange-bellied parrot | *Neophema chrysogaster* | Captive | This study |
| KF188685 | Australia | 2013 | Orange-bellied parrot | *Neophema chrysogaster* | Captive | This study |
| KF188686 | Australia | 2013 | Orange-bellied parrot | *Neophema chrysogaster* | Captive | This study |
| KF188687 | Australia | 2013 | Orange-bellied parrot | *Neophema chrysogaster* | Captive | This study |
| KF188688 | Australia | 2013 | Orange-bellied parrot | *Neophema chrysogaster* | Captive | This study |
| KF188689 | Australia | 2013 | Orange-bellied parrot | *Neophema chrysogaster* | Captive | This study |
| KF188690 | Australia | 2013 | Orange-bellied parrot | *Neophema chrysogaster* | Captive | This study |
| KF188691 | Australia | 2013 | Orange-bellied parrot | *Neophema chrysogaster* | Captive | This study |
| KF188692 | Australia | 2013 | Orange-bellied parrot | *Neophema chrysogaster* | Captive | This study |
| KF188693 | Australia | 2013 | Orange-bellied parrot | *Neophema chrysogaster* | Captive | This study |
| KF188694 | Australia | 2013 | Orange-bellied parrot | *Neophema chrysogaster* | Captive | This study |
| KF561250 | Australia | 2013 | Orange-bellied parrot | *Neophema chrysogaster* | Captive | This study |
| AF311300 | Australia | 2000 | Major mitchell’s cockatoo | *Lophochroa leadbeateri* | Captive | Bassami et al., 2001 |
| AF080560 | Australia | 1998 | Sulphur-crested cockatoo | *Cacatua galerita* | Captive | Bassami et al., 1998 |
| AF311301 | Australia | 2000 | Sulphur-crested cockatoo | *Cacatua galerita* | Wild | Bassami et al., 2001 |
| AF311302 | Australia | 2000 | Sulphur-crested cockatoo | *Cacatua galerita* | Captive | Bassami et al., 2001 |
| AY450436 | South Africa | 2003 | White cockatoo | *Cacatua alba* | Captive | Heath et al., 2004 |
| GU015022 | Thailand | 2006 | Palm cockatoo | *Probosciger aterrimus* | Captive | Sariya et al., (unpublished) |
| FJ685980 | Thailand | 2006 | Blue-and-yellow macaw | *Ara ararauna* | Captive | Sariya et al., (unpublished) |
| FJ685978 | Thailand | 2005 | Yellow-crested cockatoo | *Cacatua sulphurea* | Captive | Sariya et al., (unpublished) |
| FJ685979 | Thailand | 2005 | Yellow-crested cockatoo | *Cacatua sulphurea* | Captive | Sariya et al., (unpublished) |
| FJ685989 | Thailand | 2006 | Salmon-crested cockatoo | *Cacatua moluccensis* | Captive | Sariya et al., (unpublished) |
| AF311297 | Australia | 2000 | Eastern long-billed corella | *Cacatua tenuirostris* | Captive | Bassami et al., 2001 |
| AF311298 | Australia | 2000 | Galah | *Eolophus roseicapillus* | Captive | Bassami et al., 2001 |
| EF457974 | Australia | 2007 | Cockatiel | *Nymphicus hollandicus* | Captive | Shearer et al., 2008 |
| EF457975 | Australia | 2007 | Cockatiel | *Nymphicus hollandicus* | Captive | Shearer et al., 2008 |
| AB514568 | Japan | 2010 | Cockatiel | *Nymphicus hollandicus* | Captive | Kotah et al., 2010 |
| AY450434 | South Africa | 2003 | White-bellied caique | *Pionites leucogaster* | Captive | Heath et al., 2004 |
| AF071878 | USA | 1998 | Unknown (pooled blood) | Unknown | Captive | Niagro et al., 1998 |
| GU936288 | New Zealand | 2008 | Red-fronted parakeet | *Cyanoramphus novaezelandiae* | Wild | Massaso et al., 2012 |
| GU936293 | New Zealand | 2008 | Red-fronted parakeet | *Cyanoramphus novaezelandiae* | Wild | Massaso et al., 2012 |
| JF519618 | New Zealand | 2008 | Red-fronted parakeet | *Cyanoramphus novaezelandiae* | Wild | Massaso et al., 2012 |
| GQ396653 | New Zealand | 2008 | Red-fronted parakeet | *Cyanoramphus novaezelandiae* | Wild | Ortiz-Catedral et al., 2010 |
| GQ396654 | New Zealand | 2008 | Red-fronted parakeet | *Cyanoramphus novaezelandiae* | Wild | Ortiz-Catedral et al., 2010 |
| JF519619 | New Zealand | 2010 | Eastern rosella | *Platycercus eximius* | Wild | Massaso et al., 2012 |
| JQ782196 | New Zealand | 2010 | Eastern rosella | *Platycercus eximius* | Wild | Massaso et al., 2012 |
| GU936287 | New Zealand | 2008 | Eastern rosella | *Platycercus eximius* | Wild | Massaso et al., 2012 |
| JQ782198 | New Zealand | 2010 | Eastern rosella | *Platycercus eximius* | Wild | Massaso et al., 2012 |
| JQ782199 | New Zealand | 2010 | Eastern rosella | *Platycercus eximius* | Wild | Massaso et al., 2012 |
| JQ782197 | New Zealand | 2010 | Eastern rosella | *Platycercus eximius* | Wild | Massaso et al., 2012 |
| JQ782200 | New Zealand | 2010 | Eastern rosella | *Platycercus eximius* | Wild | Massaso et al., 2012 |
| AF311295 | Australia | 2000 | Bluebonnet | *Psephotus haematogaster* | Captive | Bassami et al., 2001 |
| AF311296 | Australia | 2000 | Rosey-faced lovebird | *Agapornis roseicollis* | Captive | Bassami et al., 2001 |
| AY521235 | United Kingdom | 2004 | Rosey-faced lovebird | *Agapornis roseicollis* | Captive | de Kloet & de Kloet, 2004 |
| FJ685985 | Thailand | 2005 | Lovebird | *Agapornis sp* | Captive | Sariya et al., (unpublished) |
| AY450442 | Zambia | 2003 | Black-cheeked lovebird | *Agapornis personata* | Captive | Heath et al., 2004 |
| AY450435 | South Africa | 2003 | African grey parrot | *Psittacus erithacus* | Captive | Heath et al., 2004 |
| GU015012 | Thailand | 2006 | African grey parrot | *Psittacus erithacus* | Captive | Sariya et al., (unpublished) |
| GU015020 | Thailand | 2006 | Eclectus parrot | *Eclectus roratus* | Captive | Sariya et al., (unpublished) |
| AY521234 | USA | 2004 | Ring necked parakeet | *Psittacula kramerii* | Captive | de Kloet & de Kloet, 2004 |
| HM748929 | South Africa | 2008 | Ring necked parakeet | *Psittacula krameri* | Captive | Varsani et al., 2011 |
| HM748927 | South Africa | 2008 | Ring necked parakeet | *Psittacula krameri* | Captive | Varsani et al., 2011 |
| AY450438 | South Africa | 2003 | Cape parrot | *Poicephalus robustus* | Captive | Heath et al., 2004 |
| DQ397818 | South Africa | 2006 | Cape parrot | *Poicephalus robustus* | Captive | Heath et al., 2004 |
| HM748918 | South Africa | 2008 | Cape parrot | *Poicephalus robustus* | Captive | Varsani et al., 2011 |
| HM748924 | South Africa | 2008 | Amazon parrot | *Amazona sp.* | Captive | Varsani et al., 2011 |
| HM748926 | South Africa | 2008 | Eclectus parrot | *Eclectus roratus* | Captive | Varsani et al., 2011 |
| JX049213 | New Caledonia | 2011 | Eclectus parrot | *Eclectus roratus* | Captive | Julian et al., 2012 |
| JX049196 | New Caledonia | 2011 | Coconut lorikeet | *Trichoglossus haematodus deplanchii* | Captive | Julian et al., 2012 |
| JX049199 | New Caledonia | 2011 | Coconut lorikeet | *Trichoglossus haematodus deplanchii* | Captive | Julian et al., 2012 |
| JX049204 | New Caledonia | 2011 | Coconut lorikeet | *Trichoglossus haematodus deplanchii* | Captive | Julian et al., 2012 |
| JX049195 | Australia | 2009 | Rainbow lorikeet | *Trichoglossus haematodus* | Captive | Julian et al., 2012 |
| AF311299 | Australia | 2000 | Rainbow lorikeet | *Trichoglossus haematodus* | Captive | Bassami et al., 2001 |
| GQ329705 | Portugal | 2009 | African grey parrot | *Psittacus erithacus* | Captive | Henriques and Fevereiro, (unpublished) |
| GU047347 | Portugal | 2009 | African grey parrot | *Psittacus erithacus* | Captive | Henriques and Fevereiro, (unpublished) |
| GQ120621 | Portugal | 2008 | African grey parrot | *Psittacus erithacus* | Captive | Henriques and Fevereiro, (unpublished) |
| EU810207 | Portugal | 2008 | African grey parrot | *Psittacus erithacus* | Captive | Henriques and Fevereiro, (unpublished) |
| EU810208 | Portugal | 2005 | African grey parrot | *Psittacus erithacus* | Captive | Henriques and Fevereiro, (unpublished) |
| AY521237 | Germany | 2004 | African grey parrot | *Psittacus erithacus* | Captive | de Kloet & de Kloet, 2004 |
| AY450443 | South Africa | 2003 | African grey parrot | *Psittacus erithacus* | Captive | Heath et al., 2004 |
| AY450441 | South Africa | 2003 | Jardine parrot | *Poicephalus gulielmi massaicus* | Captive | Heath et al., 2004 |
| HM748919 | South Africa | 2008 | Jardine parrot | *Poicephalus gulielmi massaicus* | Captive | Varsani et al., 2011 |
| HM748922 | South Africa | 2008 | Jardine parrot | *Poicephalus gulielmi massaicus* | Captive | Varsani et al., 2011 |
| HM748923 | South Africa | 2008 | Jardine parrot | *Poicephalus gulielmi massaicus* | Captive | Varsani et al., 2011 |
| GQ165757 | South Africa | 2008 | Budgerigar | *Melopsittacus undulatus* | Captive | Varsani et al., 2010 |
| GQ165756 | South Africa | 2008 | Budgerigar | *Melopsittacus undulatus* | Captive | Varsani et al., 2010 |
| AB277747 | Japan | 2006 | Budgerigar | *Melopsittacus undulatus* | Captive | Ogawa et al., 2010 |
| AB277746 | Japan | 2006 | Budgerigar | *Melopsittacus undulatus* | Captive | Ogawa et al., 2010 |
| AB277748 | Japan | 2006 | Budgerigar | *Melopsittacus undulatus* | Captive | Ogawa et al., 2010 |
| AB277749 | Japan | 2006 | Budgerigar | *Melopsittacus undulatus* | Captive | Ogawa et al., 2010 |
| AB277750 | Japan | 2006 | Budgerigar | *Melopsittacus undulatus* | Captive | Ogawa et al., 2010 |
| AB277751 | Japan | 2006 | Budgerigar | *Melopsittacus undulatus* | Captive | Ogawa et al., 2010 |
| GQ386944 | China | 2008 | Budgerigar | *Melopsittacus undulatus* | Captive | Zhuang et al., (unpublished) |
| AY521234 | USA | 2004 | Ring necked parakeet | *Psittacula kramerii* | Captive | de Kloet & de Kloet, 2004 |
| JQ782202 | New Zealand | 2012 | Yellow-crowned Parakeet | *Cyanoramphus auriceps* | Wild | Massaso et al., 2012 |
| JQ782203 | New Zealand | 2012 | Yellow-crowned Parakeet | *Cyanoramphus auriceps* | Wild | Massaso et al., 2012 |
| JQ782204 | New Zealand | 2012 | Yellow-crowned Parakeet | *Cyanoramphus auriceps* | Wild | Massaso et al., 2012 |
| JQ782205 | New Zealand | 2012 | Yellow-crowned Parakeet | *Cyanoramphus auriceps* | Wild | Massaso et al.,2012 |
| JQ782207 | New Zealand | 2012 | Yellow-crowned Parakeet | *Cyanoramphus auriceps* | Wild | Massaso et al., 2012 |
| JQ782208 | New Zealand | 2012 | Yellow-crowned Parakeet | *Cyanoramphus auriceps* | Wild | Massaso et al., 2012 |
